# Recent advances in dead cell clearance during acute lung injury and repair

**DOI:** 10.12703/r/10-33

**Published:** 2021-03-30

**Authors:** Patrick M Noone, Sekhar P Reddy

**Affiliations:** 1 Department of Pediatrics, College of Medicine, University of Illinois at Chicago, IL 60612, USA; 2 Department of Pathology, College of Medicine, University of Illinois at Chicago, IL 60612, USA

**Keywords:** Acute lung injury, Lung repair, Macrophages, Alveoli, Efferocytosis

## Abstract

Acute lung injury (ALI) and acute respiratory distress syndrome (ARDS) are clinical syndromes that cause significant mortality in clinical settings and morbidity among survivors accompanied by huge healthcare costs. Lung-resident cell dysfunction/death and neutrophil alveolitis accompanied by proteinous edema are the main pathological features of ALI/ARDS. While understanding of the mechanisms underlying ALI/ARDS pathogenesis is progressing and potential treatments such as statin therapy, nutritional strategies, and mesenchymal cell therapy are emerging, poor clinical outcomes in ALI/ARDS patients persist. Thus, a better understanding of lung-resident cell death and neutrophil alveolitis and their mitigation and clearance mechanisms may provide new therapeutic strategies to accelerate lung repair and improve outcomes in critically ill patients. Macrophages are required for normal tissue development and homeostasis as well as regulating tissue injury and repair through modulation of inflammation and other cellular processes. While macrophages mediate various functions, here we review recent dead cell clearance (efferocytosis) mechanisms mediated by these immune cells for maintaining tissue homeostasis after infectious and non-infectious lung injury.

## Acute lung injury

Acute lung injury (ALI) and its most severe form, acute respiratory distress syndrome (ARDS), together affect approximately 200,000 patients per year in the United States alone, with reported mortality rates of about 30–40%^1^. Oxygen supplementation (hyperoxia) by mechanical ventilation remains the primary therapy used for supporting critically ill patients with ALI/ARDS proven to decrease mortality^2^, but as many as 9–27% of patients undergoing this therapy contract nosocomial pneumonia, leading to excess morbidity and mortality^3^. Nosocomial pneumonia poses a substantial cost burden^4^ and accounts for approximately 27% of hospital-borne infections in American ICUs, of which 86% of cases were associated with mechanical ventilation^2^. Thus, identification of novel mechanisms underlying abnormal lung repair and microbial susceptibility may provide a basis for new therapeutic strategies that can improve clinical outcomes and decrease healthcare costs associated with ALI/ARDS.

Understanding of the mechanisms underlying ALI/ARDS is evolving, but, aside from ventilation, limited therapies of significant clinical benefit are available for intervening in lung injury progression^5,6^. Currently, treatments include statin therapy^7^, nutritional strategies^8^, and mesenchymal cell therapy^9,10^, but persistence of high mortality rates demonstrates their limitations and warrants exploration of alternative approaches^5,6^. Strategies for promoting lung repair that show favorable *in vitro* and *in vivo* results include plasma membrane repair via amphiphilic macromolecules^11^, administration of growth factors^12^, selective blockade of matrix metalloproteinases^13,14^, and modulation of proliferation-regulating transcription factors^15,16^. Additionally, gene therapy studies using viral and non-viral vector delivery, gene expression strategies, or enhanced therapeutic targeting offer promising evidence of restoring lung function, clearing inflammation, and enhancing repair mechanisms *in vitro* and *in vivo*^6^. However, clinical use of these techniques requires extensive progress to be made in terms of basic science and its translational approach. Perhaps most importantly, there are still immense gaps in our knowledge of molecular targets involved in the pathogenesis of ALI/ARDS. Therefore, better characterization of cellular mechanisms involved in heightened inflammation resolution and repair is necessary to develop novel therapies for ALI/ARDS patients.

Alveolar macrophages (AMФs) account for approximately 95% of airspace leukocytes^17^. They are major regulators of the lung inflammatory microenvironment and the first line of defense against infectious and non-infectious stimuli^18^. The course of systemic inflammation and progression to ALI/ARDS is heavily dependent on signaling from these cells, and their defective functioning is associated with multiple acute and chronic inflammatory conditions^19,20^. This regulatory capability is in part due to the phagocytic role AMФs play in clearing dead cells from the alveolar space, which facilitates injury resolution and prevents necrosis of apoptotic cells and release of pro-inflammatory mediators^21^. AMФs are highly functionally heterogeneous and phenotypically variable, which allows them to use intracellular signals to switch between pro-inflammatory and anti-inflammatory states as well as several further subdivisions and hybrid states. It is known that AMФ subtype populations vary between healthy individuals and patients with ALI/ARDS^22^, and by further investigating the cellular mechanisms by which this variation occurs it is likely we may discover new immunomodulatory targets that have the potential to mitigate the devastating effects of ALI/ARDS.

## Role of apoptosis in amplifying lung inflammatory responses and injury

The main pathological features of ALI/ARDS include alveolar epithelial and endothelial cell death, neutrophil alveolitis, and destruction of epithelial capillary barriers, leading to vascular permeability and edema infiltration^5,6^. Furthermore, hyperoxic ventilation causes excess epithelial and endothelial cell death, exacerbates pre-existing lung injury and inflammation, and impairs alveolar fluid clearance^23^. Unchecked inflammation and cell death can promote tissue scarring, organ damage, and the development of autoimmune and chronic inflammatory disorders^24^, and impaired management of these insults can have severe long-term consequences^25^. Pro-apoptotic members of the tumor necrosis factor (TNF) family, Fas/FasL, are known to facilitate cell death, and increased concentrations of these mediators have been detected in bronchoalveolar lavage (BAL) samples of ARDS patients^26,27^. Instillation of Fas/FasL induced lung injury and inflammation^28–30^, while inhibition of Fas/FasL signaling or apoptosis attenuated lung injury in animals subjected to endotoxemia and mechanical ventilation^29,30^. This suggests that apoptosis, to some extent, affects the severity of ALI/ARDS progression and how well patients recover. Apoptotic cells may undergo secondary necrosis, or unprogrammed cell death, if not removed, leading to the release of endogenous ligands called damage-associated molecular patterns (DAMPs). High-mobility group box 1 (HMGB1) and other DAMPs resemble pathogen-associated molecular patterns (PAMPs) such as lipopolysaccharide (LPS)^19^. These molecules exacerbate tissue inflammation and contribute to injury observed in ALI/ARDS, COPD, pneumonia, asthma, and pulmonary fibrosis^20^. As expected, elevated HMGB1 levels were found in BAL obtained from peripheral airways of COPD patients^31^. Many patients at risk for ALI require medical attention well into the course of their initial systemic inflammatory illness, which means that blocking late-acting DAMPS may have greater clinical relevance than more rapidly released mediators. However, it appears that while ALI may resolve entirely in some patients, along with lung function recovery, other patients are more susceptible to the development of chronic disorders^5,6^.

Pyroptosis (pyro meaning “fever” or “fire”) is a pro-inflammatory process of pre-programmed cell death distinct from apoptosis that results from activation of inflammatory proteases belonging to the caspase family, particularly caspase-1, -4, and -5^32^. Inflammasomes are multiprotein complexes assembled by pattern recognition receptors (PRRs) in response to bacterial or viral PAMPs (e.g. LPS, bacterial flagella, viral DNA and RNA) and/or damaged host-cell derived DAMPs. These complexes recruit either caspase-1 in the canonical inflammasome or caspase-4 and -5 in the non-canonical inflammasome^33^. Recruitment occurs either directly or indirectly through a caspase activation and recruitment domain (CARD) containing adapter protein called an apoptosis-associated speck-like protein containing a CARD (ASC)^32^. The activated caspase molecules serve two crucial functions: 1) proteolytic cleavage of perforation inducing protein Gasdermin D, which creates pores in the cell membrane to induce pyroptosis, and 2) cleavage of IL-1β and IL-18 into their active forms, which are then released by the pyroptotic cell and initiate a pro-inflammatory response^33,34^. Furthermore, pyroptosis also promotes HMGB1 release, which as mentioned is highly expressed in inflammatory lung conditions^30,35^. Pyroptosis works in accordance with apoptosis and is necessary for clearing pathogen-infected cells but when unrestricted can induce inflammation and can lead to organ failure, sepsis, and death^34^. In addition to pyroptosis, necrosis and necroptosis, ferroptosis, and autophagy-dependent cell death are all distinct from apoptosis in their activating stimuli^36–38^ but nonetheless must be cleared from the alveolar space to prevent ALI/ARDS.

The phagocytic machinery that recognizes dead cells is regulated by signaling cascades and selective upregulation of anti-inflammatory genes coordinated by communication between apoptotic cells and phagocytes^39^. Engulfment of apoptotic cells by phagocytosis results in an abundance of reactive oxygen species (ROS), which stimulates macrophage apoptosis and inflammation persistence^40,41^. Regarding the role phagocytes play in mitigating ALI/ARDS progression, AMФs are recognized as initiators of pro-repair and pro-resolution processes necessary for restoring lung function following injury^42^. However, AMФs also recruit inflammatory cells, produce pro-inflammatory cytokines, and mediate pro-fibrotic processes^43^. This functional dynamic makes AMФs influential during both acute and resolution/recovery phases of lung injury^44–46^. Studies performed with serial BAL in humans with ARDS determined that increased AMФ numbers and matured cellular phenotype correlated with favorable clinical outcomes^47–49^. Currently, there is increasing evidence suggesting that macrophages, including resident AMФs and recruited AMФs derived from blood-circulating monocytes, are key regulators of ALI/ARDS pathogenesis^50,51^. These macrophages are phenotypically flexible and functionally heterogeneous, suggesting a key regulatory role in inflammation, injury, and repair throughout the course of ALI/ARDS^52,53^. This dual functionality AMФs play in both resolving and inducing inflammation demonstrates their unique and evasive role in maintaining lung homeostasis. Since these processes remain incompletely understood, further investigating the role of AMФs in ALI/ARDS pathogenesis is of clinical interest.

## Macrophage plasticity and lung injury resolution

Phagocytes are classified as either “professional” or “non-professional”. Professional phagocytes (e.g. monocytes, macrophages, neutrophils, etc.) are more abundant, secrete more cytokines, and display a wider range of phagocytic receptors^54^ than their non-professional counterparts (e.g. epithelial cells, fibroblasts, etc.). They act as first responders to infection in the steady state by recognizing and removing bacteria and promote adaptive immunity by displaying antigens of digested pathogens for T and B cell recognition^55^. Phagocytes use PRRs including Toll-like receptors (TLRs), Nod-like receptors (NLRs), and RIG-I-like receptors (RLRs) to initiate phagocytosis or inflammatory signal transduction in response to microbial infection^56,57^. In the lung, macrophages comprise two subtypes: resident and recruited. The former and more prevalent population is found within the alveoli themselves, whereas the latter are derived from circulating monocytes recruited from the interstitial space to infection or injury sites^58^. Resident AMФs originate from progenitor yolk sac and fetal liver monocytes and become functionally active as soon as the first week after birth, continuously repopulating alveoli by auto-regeneration^59^. Resident AMФs act as sentinels providing the first line of defense against respiratory infection and injury by clearing pathogens and debris^60^. However, severe enough insult can induce circulating (bone marrow-derived) monocyte migration from the periphery to the inflamed tissue, where they differentiate into monocyte-derived AMФs and initiate a pro-inflammatory and profibrotic response^42,58^.

**Figure 1.**
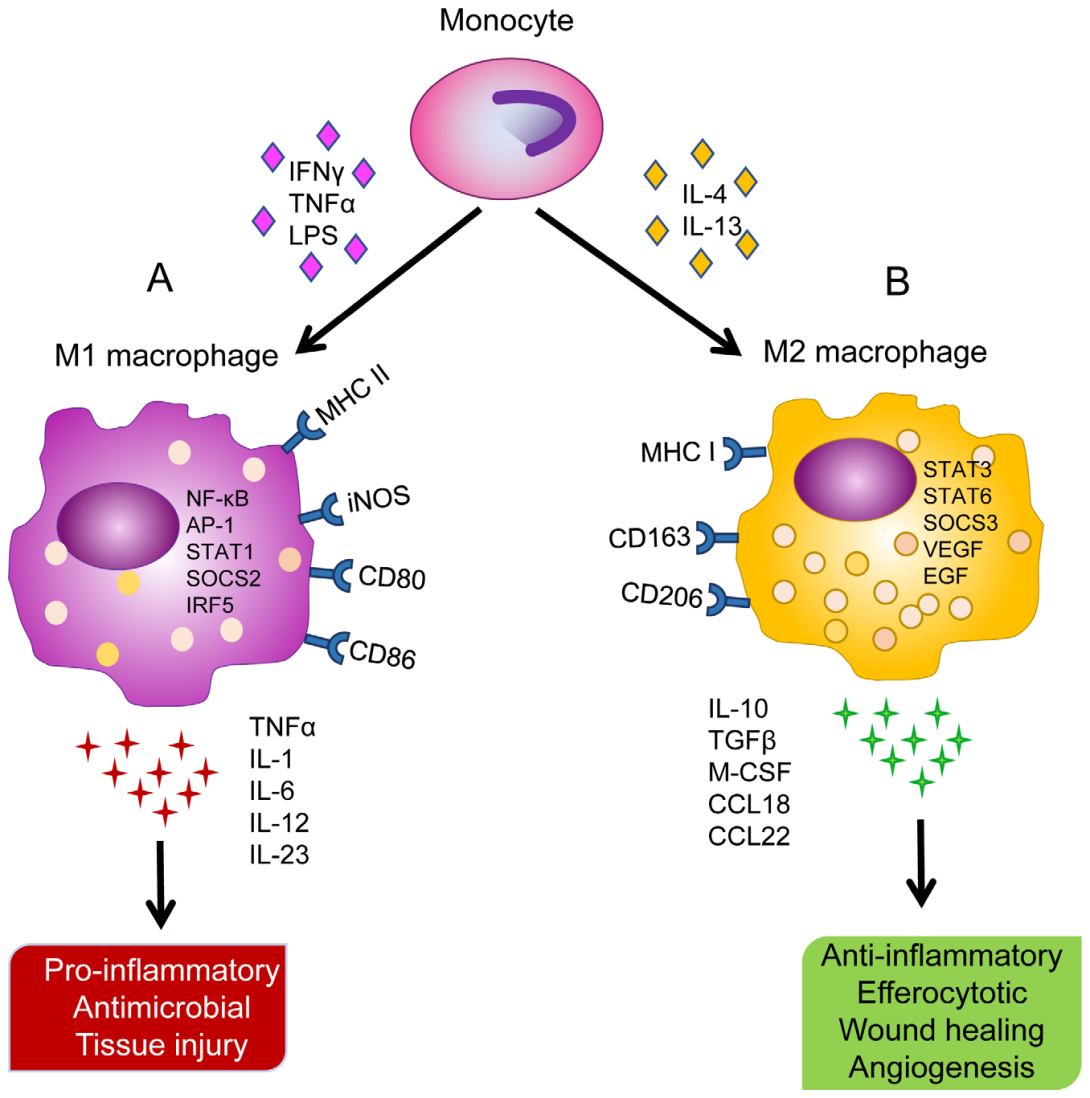
Different stimuli contributing to macrophage polarization along with differential surface markers, gene regulation, cytokine release, and physiological functions. **A**) Monocyte differentiation into classically activated alveolar macrophages (AMФ^M1^) upon stimulation with interferon gamma (IFNγ), tumor necrosis factor (TNF)-α, and lipopolysaccharide (LPS). High expression of major histocompatibility complex (MHC) II, inducible nitric oxide synthase (iNOS), CD80, and CD86 surface markers. Upregulation of nuclear factor (NF)-κB, activator protein 1 (AP-1), signal transducer and activator of transcription 1 (STAT1), suppressors of cytokine signaling 2 (SOCS2), and interferon regulatory factor 5 (IRF5). Release of TNF-α, interleukin (IL)-1, IL-6, IL-12, and IL-23 cytokines leading to pro-inflammatory response, antimicrobial activity, and collateral tissue injury. **B**) Monocyte differentiation into alternatively activated alveolar macrophages (AMФ^M2^) upon stimulation with IL-4 and IL-13. High expression of MHC I, CD163, and CD206 surface markers. Upregulation of STAT3, STAT6, SOCS3, vascular endothelial growth factor (VEGF) and epithelial growth factor (EGF). Release of IL-10, transforming growth factor (TGF)-β, macrophage colony-stimulating factor (M-CSF), CCL18, and CCL22 leading to anti-inflammatory response, efferocytosis, wound healing, and angiogenesis.

AMФs are particularly unique in their phenotypic plasticity, which refers to polarization between two distinct phenotypes depending on inflammatory microenvironment conditions^61^. Classically activated (M1) AMФs (AMФs^M1^) are cytotoxic and pro-inflammatory mediators that protect against pathogens by secreting pro-inflammatory cytokines and promoting Th1-type immunity^62^. Macrophage stimulation with interferon gamma (IFNγ) or TNF alpha (TNF-α) in accordance with TLR agonists (e.g. LPS) induces M1 polarization. AMФs^M1^ produce cytotoxic and bactericidal ROS, reactive nitrogen species (RNS), and Th1 pro-inflammatory cytokines (e.g. IL-1, IL-6, IL-12, IL-23, and TNF-α) and strongly express major histocompatibility complex (MHC) II, CD80, CD86, and iNOS surface markers^63–65^ (Figure 1A). The classically activated phenotype promotes inflammation and assists in opsonization, antibody-dependent cytotoxicity, and phagocyte-dependent defense functions. Enhanced clearance of dead cells performed by pro-resolution AMФs is a key process in tissue repair and resolution of AMФ^M1^-promoted inflammation. This process limits the production of pro-inflammatory cytokines (e.g. IFNγ, TNF-α, IL-1, IL-6, IL-8, and LTB4) and increases the production of anti-inflammatory/reparative cytokines (e.g. M-CSF, IL-4, IL-10, IL-13, and transforming growth factor beta [TGF-β])^43^.

Alternatively activated (M2) AMФs (AMФs^M2^) are anti-inflammatory and promote tissue repair, fibrotic remodeling, and Th2-type immunity^62,66^. IL-4 and IL-13 play an important role in resolving inflammation and aid in lung regeneration by facilitating wound healing through suppression of inflammatory signaling (Figure 1B). Macrophage stimulation with IL-4 and IL-13 induces AMФ^M2^ polarization, leading to the resolution of lung inflammation. AMФ phenotypic changes are largely regulated by signal transducer and activator of transcription (STAT) transcription factors and suppressors of cytokine signaling (SOCS). STAT1/SOCS2 activation promotes the AMФ^M1^ phenotype while activation of STAT3/6 and SOCS3 promotes the AMФ^M2^ phenotype^67^. AMФs^M2^ have been shown to resolve inflammation and initiate wound healing via the production of immunosuppressive cytokines (e.g. IL-10, TGF-β, CCL18, and CCL22) and angiogenesis mediators (e.g. VEGF and EGF) as well as express high levels of scavenger receptors (e.g. CD163 and CD206)^63–65^. AMФs^M2^ also release immunosuppressive cytokines such as TGF-β, an inhibitor of NO production^63^, and Arginase 1, which neutralizes reactive nitrogen intermediates^50,68^. AMФs^M2^ are highly heterogeneous, with several further subdivisions (i.e. M2a, M2b, and M2c)^61,69^. Distinct functional roles between AMФs^M1^ and AMФs^M2^ suggest that a counterbalance between their pro- and anti-inflammatory responsibilities must be maintained to promote lung homeostasis in response to infection and injury.

M1 and M2 classification is generally useful for describing functional differences in AMФs throughout inflammatory processes but as a dichotomy neglects what appears to be a continuum of activation states that exists *in vivo*^70^. AMФs are constantly altered by extrinsic factors, with M1 and M2 phenotypes representing the extreme sides of an expression spectrum^71^. In fact, most AMФs in the steady state display markers of both M1 and M2 phenotypes simultaneously, which is thought to allow quick switching between M1 and M2 functions^72^. Due to this flexible programming, AMФs have shown critical activity at all stages of alveolar repair and fibrosis and phenotype-dependent roles at distinct inflammatory and resolving phases. Transcriptomic datasets have provided immense amounts of information regarding macrophage integration and computation of local inflammatory signals, and understanding of AMФ transcriptional regulation can potentially be used to locate macrophage subset-specific therapeutic targets^73^. Flexibility in AMФ programming and their adaptability to environmental changes suggests modulating these processes may provide therapeutics for ALI/ARDS patients.

## Neutrophil death contributions to ALI/ARDS

Neutrophils are specialized leukocytes with life cycles ranging from only a few hours to days^61^. Due to these cells’ short lifespans, neutrophil death is highly concerted and can occur via several mechanisms, including apoptosis, necrosis/necroptosis, and release of neutrophil extracellular traps (NETs). Neutrophil alveolitis and cell death contribute to inflammatory injury observed in ALI/ARDS patients, and their activating stimuli leading to efferocytosis influence the course of systemic inflammation. Distinct modes of neutrophil cell death have been implicated in several pathologies, including cancer, neurodegenerative disease, and autoimmune disorders^74^. Neutrophils are recruited as first responders to microbial infection to fight off invading pathogens, where they participate in phagocytosis, degranulation, ROS release, and NET release^74^. NETs are DNA–protein complexes released by neutrophils to neutralize pathogens in a process called NETosis. NETs are increasingly being investigated as contributors towards ALI/ARDS^75,76^. *In vitro*, *in vivo*, and clinical studies have confirmed that NETs promote ARDS inflammation by inducing AMФ^M1^ polarization and pro-inflammatory cytokine release, and increased M1 markers and decreased M2 markers were found in ARDS BAL fluid and lung tissue^77^. Furthermore, ARDS patients experience increased NET formation accompanied by decreased levels of AMФ engulfment of NETs and apoptotic neutrophils^78^. Neutralization of HMGB1 in the BAL fluid was shown to improve efferocytosis and NET clearance^78^, and engulfment of apoptotic neutrophils by phagocytes was found to promote anti-inflammatory signaling and homeostasis maintenance^79^. These results demonstrate that neutrophil contributions to ALI/ARDS are at least in part due to their influence on AMФ phenotype switching and in part due to the effectiveness of efferocytotic clearance following cell death.

## Efferocytosis and resolution of inflammatory lung injury

Host defense and the protective roles leukocyte recruitment and phagocytosis play in acute inflammatory injury were first described in 1908 by Nobel Prize laureate Elie Metchnikoff^80^. However, much remains unclear as to how cellular communication facilitates apoptotic cell clearance and promotes homeostasis. As many as 150 billion cells, representing 0.4% of the body’s cellular mass, are known to be turned over via apoptosis every day in the average adult^81^. Apoptotic cells are rarely observed, even in tissues with frequent cell turnover^82^, which suggests an efficient framework for clearing dead cells^24^. Removal of apoptotic cells and debris by phagocytosis, a term coined “efferocytosis” by Henson, Gardai *et al*. (from the Latin *effero* meaning “to carry to the grave” or “to bury”)^83,84^, appears to serve a crucial protective role against inflammatory injury. The process by which dead cells are identified, taken up, and disposed of by phagocytes is a highly regulated and concerted series of coordinated signaling (see below).

## Activation of efferocytosis machinery

**Figure 2.**
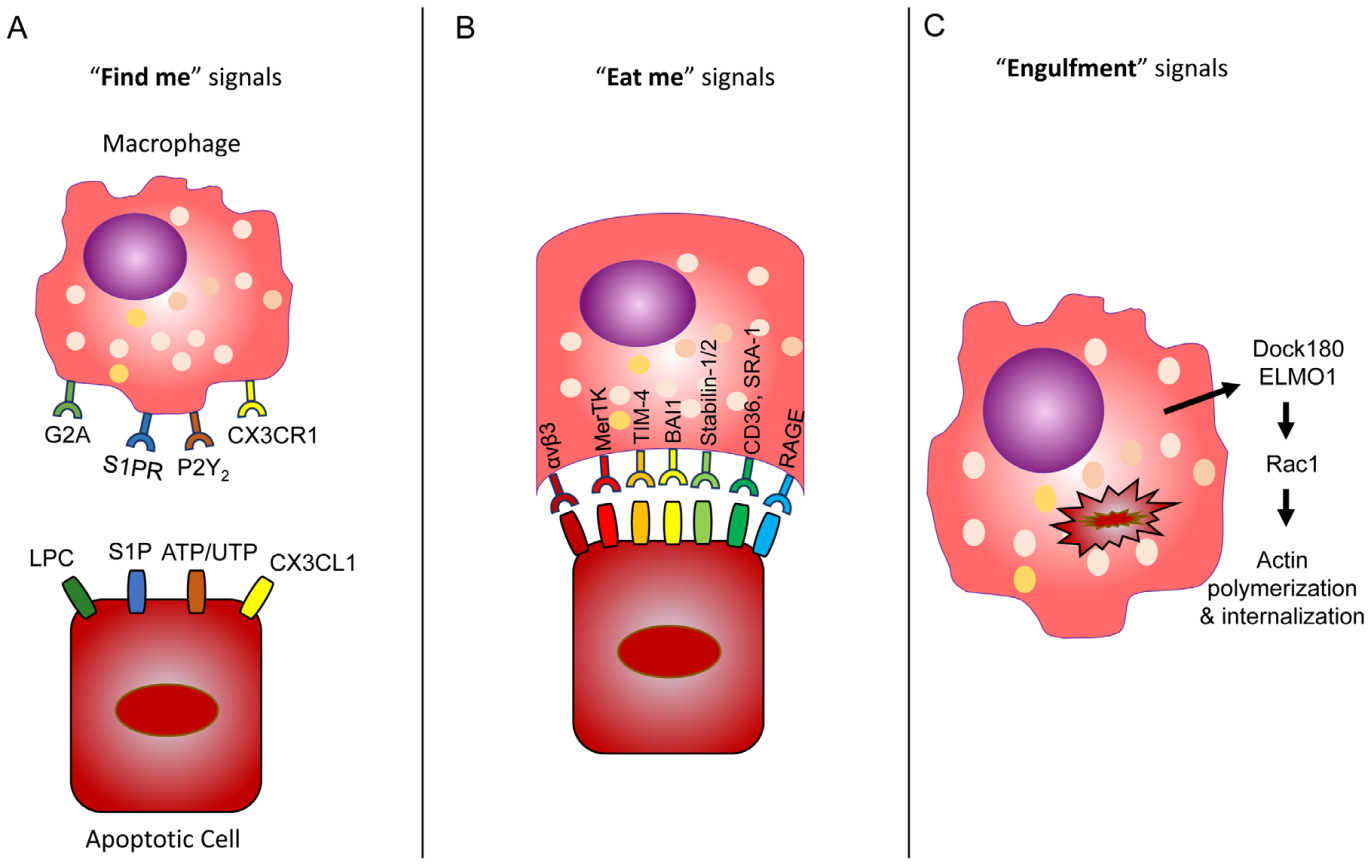
Efferocytosis broken down into “find me”, “eat me”, and engulfment signals. **A**) Apoptotic cells express “eat me” signals including lysophosphatidylcholine (LPC), sphingosine-1-phosphate (S1P), nucleotides ATP/UTP, and CX3CL1. These signals are recognized by G2A, S1PR, P2Y_2_, and CX3C motif chemokine receptor 1 (CX3CR1) receptors, respectively, by proximal macrophages. **B**) Recognized cells use Ptd-Ser receptors as an “eat me” signal to initiate engulfment, which can include αvβ3 integrins, MerTK, TIM-4, BAI1, stabilin-1/2, CD36, SRA-1, and receptor for advanced glycation end products (RAGE). **C**) Following engulfment, dedicator of cytokinesis protein 1 (Dock180) and engulfment and cell motility protein 1 (ELMO1) act together as a guanine nucleotide exchange factor (GEF) to induce Rac1 GTPase, leading to cytoskeletal changes via actin polymerization followed by apoptotic cell internalization and phagolysosomal degradation.

Efferocytotic signaling refers to phagocyte recruitment (“find me”), engulfment (“eat me”), and “post-engulfment” signals (Figure 2), and communication of these signals depends on phagocyte/apoptotic cell type, apoptotic stimuli, and stage of apoptosis^24^. Apoptotic cells release “find me” signals to initiate phagocytic uptake. Four different apoptotic “find me” signals have been identified: lysophosphatidylcholine (LPC), sphingosine-1-phosphate (S1P), nucleotides ATP and UTP, and CX3CL1 or fractalkine^39^. The first three mechanisms are caspase-3 dependent: 1) phosphatidylcholine is converted into LPC by apoptotic cells and subsequently released and recognized by G2A receptors on proximal macrophages^85^; 2) S1P, produced by the sphingosine kinase-catalyzed conversion of sphingosine, is released from apoptotic cells and recognized by S1P receptors on macrophages^86^; and 3) apoptotic release of nucleotides ATP and UTP induces monocyte recruitment through recognition by phagocytic purinergic receptors. Furthermore, ATP and UTP receptor P2Y_2_ deficiency in mice showed a significant decrease in monocyte and macrophage recruitment, and nucleotide deficiency/P2Y_2_ interference also resulted in inadequate clearance of apoptotic thymocytes^87^. In a caspase-3-independent mechanism, CX3CL1, or fractalkine, a membrane-associated protein released by apoptotic cells, binds to CX3C motif chemokine receptor 1 (CX3CR1) on phagocytes to promote recruitment^88^. Additionally, upregulation of several solute carrier (SLC) proteins was found to take place at distinct “find-me” and “eat-me” stages of efferocytosis, suggesting a complex and incompletely understood regulatory system that warrants further investigation^89^. After recognition of these apoptotic “find-me” signals, phagocytes use additional cell signaling mechanisms to dispose of marked cells.

Apoptotic cells expose phosphatidylserine (Ptd-Ser) as an “eat-me” signal that can be recognized by several receptors (Figure 2). Remarkably, many molecules have been shown to act as Ptd-Ser receptors, including scavenger receptors (CD36 and SRA-1), αvβ3 integrins, MerTK, Tim-4, BAI1, and stabilin-1 and -2^90^. Many Ptd-Sers recognize multiple ligands and likely have roles other than apoptotic cell clearance^91^. Among the known Ptd-Ser receptors, Tim-4 and BAI1 and stabilin-2 directly bind to Ptd-Ser on apoptotic cells. Tim-4 is exclusively expressed on professional phagocytes^92^ and the main receptor mediating the phagocytosis of apoptotic cells^93^. Tim-4 is thought to act as a tethering receptor rather than directly transmitting engulfment signals^94^, and Tim-4-dependent efferocytosis depends on the activation of integrins, focal adhesion kinase (FAK), and phosphoinositol-3 kinases^93^. Dysregulation of Tim-4 expression has been found in autoimmune conditions^95,96^, and expression of Tim-4 decreased in response to oxidative stress^97^. Increased expression of Ptd-Ser receptor MerTK was found in airway macrophages of cigarette smokers and has been implicated in apoptotic cell buildup in the lungs of patients with COPD^98^. The MerTK–ERK pathway is also known to play a role in the resolution of inflammation^99^. Ptd-Ser receptor Axl is thought to play a role in apoptotic cell removal and was found to be expressed in mouse airway macrophages but not in interstitial macrophages or other lung leukocytes^100^. Receptor for advanced glycation end products (RAGE) is a recently characterized Ptd-Ser receptor highly expressed in AMФs. RAGE-deficient macrophages showed impaired phagocytic uptake of apoptotic thymocytes and neutrophils and led to increased alveolar accumulation of inflammatory cells following LPS stimulation^101^. Additionally, calreticulin (CRT) is thought to act as a Ptd-Ser-binding bridging molecule that can behave as an “eat-me” signal in cell death induced by endoplasmic reticulum (ER) stress. Protein kinase RNA-like ER kinase (PERK) phosphorylates eIF2α, promoting Bap31 cleavage and Bax activation in a caspase-8-dependent manner. CRT then moves from the ER to the Golgi apparatus and is displayed by apoptotic cells through SNARE-mediated exocytosis, where recognition of low-density lipoprotein receptor-related protein (CD91) by local phagocyte receptors leads to dead cell engulfment^102^. Hodge *et al*. have done extensive work investigating efferocytotic deficiency in COPD patients and have found reduced CD31, CD91, CD44, and CD71 expression and enhanced Ki-67 expression in the lungs of smokers compared with non-smokers^103^.

In contrast to “eat me” signals, “don’t eat me” signals are displayed by healthy cells to prevent uptake by phagocytosis. Some of these signals have recently been characterized and are now of clinical interest as potential therapeutic targets in ALI/ARDS recovery. One of these “don’t eat me” signals is integrin-associated protein (CD47), a surface membrane protein activated by activator protein 1 (AP-1) transcription factor c-Jun in fibroblasts, overexpression of which is associated with fibrotic injury^104^. Interestingly, antibody-mediated blockage of CD47 was found to be sufficient for reversing fibrosis and improving lung function in mice by increasing phagocytosis of profibrotic fibroblasts^104^. CD47 is an anti-phagocytic molecule that was found to be constitutively expressed in certain myeloid leukemias, indicating its role in assisting cancer cells by evading phagocytic recognition^105^. Preclinical data on anti-CD47 cancer therapy is promising^106,107^, and clinical trials have shown optimistic results in ameliorating tumor growth^108,109^. Similarly, platelet endothelial cell adhesion molecule (CD31) is another surface membrane protein that plays a role in preventing engulfment by a repulsive CD31–CD31 interaction between healthy cells and phagocytes^110^. Many “don’t eat me” signals with therapeutic potential for alleviating ALI/ARDS remain undiscovered or are not fully understood, so continuous investigation into these molecules is of clinical interest.

## Efferocytosis-induced intracellular signaling

Following engulfment, phagocytic cytoskeletons must adapt to internalize dead cells. The Rho family of GTPases is an established regulatory factor in cellular movement and cytoskeletal changes^111^ and is involved in virtually all actin-dependent processes including mobility, adhesion, and phagocytosis^112^. Rho GTPases use guanine nucleotide exchange factors (GEFs) to switch between inactive, or GDP-bound, and active, or GTP-bound, states^112^. One of these Rho family proteins highly expressed in macrophages^113^, Rac1, induces plasma membrane remodeling to allow phagosome internalization of dead cell particles by stimulating actin polymerization via the Rac-WAVE-Arp2/3 pathway^114,115^. Rac1 activation occurs when engulfment and cell motility protein 1 (ELMO1) and dedicator of cytokinesis protein 1 (Dock180) work coactively as a GEF for Rac1, promoting the cytoskeletal changes required for internalization^116^. Additionally, the intracellular domain of BAI1 was found to interact with ELMO1 and Dock180 GEF processes^117^. The role of ELMO1/Rac1 signaling in proper inflammatory functions is becoming particularly clear. For example, ELMO1 and Rac1 were found to be necessary for internalization processes and promoting inflammatory signaling, and inhibition of ELMO1 led to a sixfold decrease in *Salmonella* internalization^118^. Furthermore, ELMO1-deficient macrophages experienced reduction in TNF-α and monocyte chemoattractant protein 1 (MCP-1) release and in nuclear factor-κB (NF-κB) activation and bacterial internalization^117,118^. Rac1 actin polymerization abilities likely involve interplay with ERK, FAK, AKT, and STAT6 as well^119^. Other members of the Rho GTPase family including Rac2, Rho, and Cdc42 showed involvement in macrophage efferocytosis^120^. Rho A is known to antagonize Rac1-mediated actin reorganization^121,122^, and its suppression assisted in apoptotic engulfment, whereas its overexpression inhibited phagocytic uptake^123^. Cigarette smoke has been found to inhibit efferocytosis through oxidant-dependent activation of RhoA, but antioxidant supplementation prevented this effect, leading to the reversal of efferocytotic impairment^124^. RhoA was found to assist in actomyosin cytoskeleton contractions via the Rho-associated coiled-coil-containing protein kinase (ROCK) pathway, as well as in other processes including cell proliferation and migration^125^. C-type lectins like mannose-binding lectin (MBL) have also shown therapeutic potential. MBL promotes apoptotic cell uptake by increasing Rac1/2/3 expression and is reduced in airways of COPD patients^126^.

## Oxidant stress and efferocytosis impairment

The accumulation of ROS inflicts intracellular destruction and initiates enhanced pro-inflammatory gene expression and cell death^127^. Engulfment of apoptotic cells creates an abundance of harmful ROS, and macrophage functions are heavily regulated by ROS production^40,41^. ROS are involved in macrophage polarization, functional and phenotypic regulation, and cell death, proliferation, and phagocytic ability^40^. ALI/ARDS patients exhibit significant oxidative stress on the lungs due to ventilation therapy-induced ROS accumulation^128^. Mitigation of excessive ROS and maintenance of intracellular redox homeostasis is crucial for ALI resolution. Cells have intrinsic antioxidant defense mechanisms for maintaining equilibrium in response to excess ROS generation^129,130^. A better understanding of the host defense response, such as antioxidant enzyme functions and deregulation in ARDS patients with and without hyperoxic ventilation, can help detect therapeutic targets that may prove useful for alleviating lung injury.

Hyperoxic stress and ROS accumulation can lead to functional changes in AMФs. For example, ROS production upregulates the expression of AMФ^M1^-associated pro-inflammatory transcription factors AP-1 and NF-κB^131^. Additionally, TNF-α is known to induce activation of NF-κB and AP-1, both of which in accordance produce inflammatory signals used by AMФs for communicating the inflammatory response to other cells in the lung^132^. The *TNF-α* promoter region was found to contain both NF-κB and AP-1 binding sites, allowing for autoregulation^133^. Glucocorticoids inhibit NF-κB and impair binding of AP-1, leading to a decrease in pro-inflammatory cytokine production, but administration of these drugs can detrimentally dampen the immune response to acute injury, making them oftentimes more harmful than useful to ALI/ARDS patients^134^. Another transcription factor, IFN regulatory factor 5 (IRF5), polarizes macrophages toward the pro-inflammatory phenotype (M1)^135^ and was found in neutrophils and other myeloid cells^136^. Blockage of IRF5 is being investigated as a therapeutic measure for alleviating inflammation^137^ and promoting efferocytosis^138^. Another IFN-regulated transcription factor is STAT1, a member of the STAT protein family. STAT1 is known to activate quickly in response to IFNs and other pro-inflammatory cytokines and leads to mitochondrial stress, ROS accumulation, and apoptosis^139^. STAT1 has been shown to modulate intracellular oxidative stress in macrophages through a p38 MAPK/STAT1/ROS positive feedback loop^140^, and the absence of NADPH oxidase (NOX)-derived superoxide in AMФs^M1^ was shown to reduce both STAT1 and IRF5 expression as well as increase AMФ^M2^ transcriptional profile^141^. ROS accumulation and inflammatory signaling are intimately related, and a better understanding of their genetic basis and how it relates to the AMФ protective role in efferocytosis can potentially reveal therapeutic targets to assist recovery in ALI/ARDS patients.

## Activation of efferocytosis to accelerate lung injury resolution

Imbalance between apoptotic cell death and efferocytosis can promote pathological conditions such as ALI/ARDS, COPD, cystic fibrosis, and asthma^142,143^, and impaired efferocytosis is implicated in complications associated with these conditions^144^. Inflammation in these lung diseases appears to worsen with inefficient removal of dead cells and debris, thus prolonging inflammation and impeding tissue repair. Delayed efferocytosis can cause apoptotic cells to undergo secondary necrosis and release DAMPs, which further promote inflammation by stimulating both innate and adaptive immune responses^20^. Efferocytotic impairment of airway macrophages leads to apoptotic and necrotic cell buildup, DAMPs release, upregulation of pro-inflammatory genes, and production of autoreactive T cells and B cells, all of which contribute to autoimmunity and chronic inflammation^145^. Prolonged inflammation can weaken resolution and potentially develop into fibrosis or chronic inflammatory conditions such as COPD^145^. Efferocytosis promotes lung homeostasis, facilitates resolution of apoptotic cell-induced inflammation^142^, and corresponds with improved clinical outcomes in ALI/ARDS patients^20^.

Although defective clearance of apoptotic cells in the development of ALI/ARDS has been proposed^20,142^ and oxidant stress affects efferocytotic ability of macrophages *in vivo* and *in vitro*^146,147^, the exact mechanisms contributing to dysfunctional efferocytosis are not completely understood. Further characterizing the molecular mechanisms used by AMФs to perform efferocytosis and resolve excessive inflammation in the lung, and how efficient functioning of these mechanisms can aid ALI/ARDS patients and other lung diseases in recovery, needs to be explored. It is possible that modulating efferocytosis can serve as a vital cellular strategy for managing the inflammatory response to injury and preventing development into chronic inflammatory disease^81,82^. Because of airway macrophages’ tissue- and disease stage-specific roles, elucidation of their efferocytotic signal activation offers promising clinical potential for better prognosis in ALI/ARDS patients with fewer off-target effects.

In summary, efferocytosis plays crucial roles not only during development and in maintaining tissue homeostasis but also during tissue repair processes through a highly regulated and concerted network of signaling. Understanding this signaling by macrophages is of clinical interest to enhance lung tissue repair and restore respiratory functions following microbial and non-microbial insults. Defining whether there are distinct and specific set(s) of macrophages that exist in the lung that carry out efferocytosis in an injury- and disease-specific manner, and how and which efferocytosis machinery is activated or affected/impaired in acute clinical syndromes resulting in chronic lung diseases, may offer better clinical prognosis and therapeutic treatment strategies. Identifying both the activators and the effectors of efferocytosis that can be easily and preferentially targetable with fewer off-target effects, perhaps by administration of small molecules/drugs (protein or non-proteinous), is necessary to optimally accelerate lung tissue repair in ALI/ARDS patients and for improving clinical outcomes and reducing huge healthcare costs associated with microbial- and non-microbial-induced lung injury.
